# The Effects of Different Exercise Approaches on Attention Deficit Hyperactivity Disorder in Adults: A Randomised Controlled Trial

**DOI:** 10.3390/bs13020129

**Published:** 2023-02-02

**Authors:** Larisa M. Dinu, Samriddhi N. Singh, Neo S. Baker, Alexandra L. Georgescu, Bryan F. Singer, Paul G. Overton, Eleanor J. Dommett

**Affiliations:** 1 Department of Psychology, Institute of Psychiatry, Psychology & Neuroscience, King’s College London, London SE5 8AF, UK; 2 School of Psychology, Sussex Addiction Research & Intervention Centre, Sussex Neuroscience, University of Sussex, Brighton BN1 9RH, UK; 3 Department of Psychology, The University of Sheffield, Cathedral Court, Sheffield S1 2LT, UK

**Keywords:** physical activity, aerobic, non-aerobic, treatment, intervention

## Abstract

Attention deficit hyperactivity disorder (ADHD) results in significant functional impairment. Current treatments, particularly for adults, are limited. Previous research indicates that exercise may offer an alternative approach to managing ADHD, but research into different types of exercise and adult populations is limited. The aim of this study was to examine the effects of acute exercise (aerobic cycling vs mind-body yoga exercises) on symptoms of ADHD in adults. Adults with ADHD (*N* = 82) and controls (*N* = 77) were randomly allocated to 10 min of aerobic (cycling) or mind-body (Hatha yoga) exercise. Immediately before and after exercise, participants completed the Test of Variables of Attention task, Delay Discounting Task, and Iowa Gambling Task to measure attention and impulsivity. Actigraphy measured movement frequency and intensity. Both groups showed improved temporal impulsivity post-exercise, with cycling beneficial to all, whilst yoga only benefited those with ADHD. There were no effects of exercise on attention, cognitive or motor impulsivity, or movement in those with ADHD. Exercise reduced attention and increased movement in controls. Exercise can improve temporal impulsivity in adult ADHD but did not improve other symptoms and worsened some aspects of performance in controls. Exercise interventions should be further investigated.

## 1. Introduction

Attention deficit hyperactivity disorder (ADHD) is a neurodevelopmental condition characterised by developmentally inappropriate levels of inattention, impulsivity and hyperactivity [1]. It affects around 5% of children [2,3] with approximately two-thirds continuing to experience symptoms as adults [4]. The continuation of childhood symptoms, combined with the possibility of adult-onset [5,6], gives an estimated adult prevalence of 2–5% [7]. The impact of ADHD is significant: it is associated with poorer academic outcomes, social difficulties, lower occupational status and substance abuse disorders [8,9]. Subsequently, the condition results in significant functional impairment [10] and reduced quality of life [11]. 

Despite the prevalence and impact of ADHD, treatments are limited. Psychostimulants are the most effective treatment and the first-line treatment for adults, reducing symptoms in 80% of patients [12,13,14]. However, they must be taken continuously to achieve this effect [15] and are associated with side effects ranging from insomnia to tachycardia [16]. Furthermore, some patients experience as little as 30% symptom reduction, meaning substantial impairments remain [17]. There are also concerns that the drugs may be abused [18,19] and 30% of adults report misuse of their medication [20]. Non-stimulants offer an alternative but with a lower response rate [21]. These drugs still have side effects including nausea and mood swings [16]. Given the limitations of these treatments, it is unsurprising that interest in alternative approaches is high. 

One alternative that has received increased attention is exercise. Exercise can take many different formats, including aerobic and non-aerobic exercises; the former refers to exercise that increases heart rate, stimulates sweating and leaves someone out of breath, whilst the latter does not [22]. Examples of aerobic exercises are running and cycling, in contrast to non-aerobic exercises which include mind-body approaches such as yoga and tai chi. Both are associated with physical and cognitive gains in healthy individuals [23,24]. The benefits of exercise may arise through several mechanisms, but there is evidence to suggest that exercise has similar effects to psychostimulants by affecting dopamine levels. For example, aerobic exercise increases dopamine levels [25], and alterations in dopaminergic markers have been found in animal models of ADHD after exercise [26]. In addition, several studies have now reported increased dopamine, albeit peripheral, after meditation or yoga in healthy adults [27,28]. The fact that exercise can increase dopamine levels adds credibility to the claim that it may be an effective treatment for ADHD. 

Although early research into the effects of exercise in ADHD tended to use correlational designs, which prevent conclusions about causality [29,30], more recent work uses experimental designs with a range of outcome measures, including ADHD symptom scales and tests of executive function such as the Flanker Test, Continuous Performances Task and various measures of memory and cognitive flexibility. It is noteworthy that whilst measures of attention and response inhibition are relatively common, few studies have examined hyperactivity. A small number of studies have examined motor skills [31,32], but only one study has measured general movement with an accelerometer after an exercise intervention [33]. Moreover, whilst different measures of attention have been used, there are no studies that consider the multidimensionality of impulsivity [34]. Furthermore, most studies focus on children and adolescents, with little research studying adults [22].

Despite these limitations, several reviews and meta-analyses have been conducted, which indicate that there may be some benefit of exercise in children, at least. For example, Neudecker et al. [35] reviewed 21 studies examining children with ADHD, with seven studies focused on acute exercise programmes and 14 focused on long-term interventions. Their work revealed that most studies used aerobic exercise, and that whilst positive effects were seen in some studies on attention and response inhibition, there was a huge variation in effect sizes; many studies used small sample sizes, with the largest including just 42 children with ADHD. As such, they concluded that larger, controlled studies were essential [35]. Similar conclusions were made by Christiansen and colleagues, who noted that more exploration of different exercise parameters was needed [36]. Critically, although these reviews have noted some positive effects of exercise in children with ADHD, the effects are not always consistent across cognitive domains; for example, positive effects have been noted in inhibition and working memory but not set-shifting [22,37,38]. This is perhaps unsurprising given that one feature of neurodivergence is diversity within an individual’s cognitive abilities, rather than simply differences between neurotypical and neurodivergent individuals [39]. 

As indicated above, the literature to date has focused on aerobic exercise. Research into the effects of non-aerobic exercise, including mind-body exercise is lacking. For example, for children with ADHD, one study reported reductions in hyperactivity and inattention after tai chi [40], and another reported improvements in attention after a walk in rural but not urban areas [41]. In both cases, additional variables were uncontrolled, making it difficult to draw firm conclusions. Similarly, two small studies investigating the effects of yoga on children produced inconsistent results [42,43]. Studies on adults are even more sparse, with a handful of small-scale studies examining tai chi [44], yoga [45] and whole body vibration [46]. The work by Converse et al. [44] and Fritz and O’Connor [47] were feasibility trials and not sufficiently powered to find treatment outcome effects; whilst Fuermaier et al. [46] did find an improvement in attention, the sample was very small. 

Despite the focus on aerobic exercise, duration of interventions has varied in previous research. Some studies have looked at long-term exercise spanning several weeks [32,48,49] but others using acute exercise indicate that as little as a five-minute run [50], five minutes of jumping [51] and ten minutes of cycling on a stationary bike [52,53] can have positive effects on ADHD-related behaviours. Furthermore, a recent meta-analysis concluded that the impact of exercise may be unaffected by intensity of exercise, age and gender [54]. Moreover, the effects also appear unaffected by whether the individual is receiving psychostimulants [55]. 

In summary, reviews in this area have called for larger, controlled studies [35,36,37,38,56], and several have noted a need for more work in adults in particular [22,57], fuller coverage of hyperactivity and impulsivity and more research looking at non-aerobic exercise [22,35]. Although adults with ADHD are underrepresented in exercise studies, limited research suggests that aerobic exercise may improve attention and processing speed [58] and response inhibition [59] as well as mood and motivation [33]. As indicated above, the small-scale trials on non-aerobic interventions using mind-body exercise have not yet produced robust findings. Furthermore, adults with ADHD have fewer treatment options available, and a recent study indicates that they would be willing to engage in exercise to manage ADHD [60], meaning this cohort warrants further investigation. Therefore, the aim of this study is to examine the effect of aerobic (cycling) and non-aerobic (Hatha yoga) exercise in adults with ADHD, using a controlled trial design to assess the impact of exercise on all symptom domains of ADHD: attention, impulsivity and hyperactivity.

## 2. Materials and Methods

### 2.1. Trial Design

This study was a randomised controlled trial with pre- and post-exercise measures in both participants with a diagnosis of ADHD and those without. Participants without ADHD were included in the study to act as a reference group and to establish whether the same effects were found in those without the condition, given that the previous literature has shown benefits of exercise on ADHD-related constructs in healthy individuals. The study protocol was registered on the ISRCTN registry for clinical trials (ID ISRCTN39271564).

### 2.2. Participant Recruitment

Participants with and without ADHD were recruited from the community through posters across university campuses, social media, institutional recruitment emails and advertisements on support groups’ websites and newsletters (e.g., UK Adult ADHD Network). All advertisements contained a link to the study information sheet and consent form where interested individuals could provide consent and complete an online screening survey. Recruited participants attended a laboratory in London to complete the study. The entire laboratory visit took around 2 h to complete. Participants were provided with a ‘thank you’ shopping voucher worth £22 (set at the London Living Wage).

### 2.3. Ethics

This research was approved in advance by the Institutional Ethics Committee (MOD-19/20-13264). All participants had access to the information sheet and an online consent form, which they were able to work through at their own pace. Additionally, consent was obtained again on arrival at the laboratory. The study was also approved through the NHS South London and Maudsley (SLaM) Trust (IRAS 279417, REC 20/NS/0053). However, due to the COVID-19 pandemic preventing typical service use, no participants were recruited via this route.

### 2.4. Eligibility Criteria for Participants

#### 2.4.1. Inclusion Criteria

All participants were aged 18–35 years old. The lower age limit was selected given the target population was adults as opposed to children or adolescents. The upper age limit was set to reduce the risk of age-related cognitive decline impacting outcomes [61], and to increase the likelihood of fitness to exercise. Additionally, all participants had to demonstrate that they were physically fit enough to exercise safely by providing positive responses to the 7-item Physical Activity Readiness Questionnaire (PAR-Q) included in the online screening survey. The PAR-Q was initially designed for the general population but is recommended for use in assessment of exercise for mental health [62] and has been previously used in those with ADHD [63,64]. Additionally, upon arrival at the laboratory, blood pressure was checked to ensure that it was within the healthy range (<140/90 mmHg). Healthy control (HC) participants were required to be free from any physical, neurological, or psychiatric conditions, and learning differences (e.g., dyslexia). They also had to score less than 14 on the Adult ADHD Self-Report Scale, screener items, ASRS-A [65]. Participants with ADHD were required to have an existing diagnosis of ADHD made by a clinician and a score of 14 or more on the ASRS-A in the screening survey. They also had to be free from any other physical or neurological condition and learning differences. Participants with ADHD were included if they were either unmedicated, and had been so for at least three months, or were receiving psychostimulant medication, and had been stable on this medication for at least one month with at least 70% adherence, as assessed by the screening survey using a previously used adherence scale [66]. Given that as many as 80% of adults with ADHD will experience co-existing psychiatric conditions, commonly depression and anxiety [67], it was not deemed appropriate to exclude all comorbidities. As such, those with depression and anxiety alongside their ADHD were eligible, provided they were not currently receiving medication for the depression or anxiety. All other comorbidities were excluded. In addition to these criteria, participants had to abstain from consuming caffeinated drinks or alcohol on the day of testing and to avoid nicotine (including e-cigarettes) for 3 h prior to testing.

#### 2.4.2. Exclusion Criteria

Participants were excluded if they were not fit enough to exercise safely, either at screening stage (PAR-Q) or at the laboratory visit (blood pressure). Those with ADHD were excluded if they did not have a set pattern of medication use, which makes it impossible to determine adherence, or if they were prescribed non-stimulant medication or other psychoactive substances either alone or in combination with psychostimulants. We excluded non-stimulant medication because the mechanism of action differs from stimulants and the proposed action of exercise. Although comorbid depression and anxiety were accepted, other neurodevelopmental (e.g., Autism Spectrum Disorder) or psychiatric conditions (e.g., bipolar disorder) were considered exclusion criteria.

### 2.5. Procedure

#### 2.5.1. Online Screening

As stated, all prospective participants were directed to an online information sheet, consent form and screening survey. The survey included items to assess inclusion criteria, including age, fitness to exercise safely (PAR-Q), ADHD symptomology (ASRS), medication use and adherence and comorbidities. In addition, demographic data were collected (gender, handedness, years in post-compulsory education) along with activity levels. The latter was measured with the Godin Leisure-Time Exercise Questionnaire [68], a three-item questionnaire in which participants state how often they engage in strenuous, moderate and light exercise over a normal seven-day period. The items are accompanied by examples of what constitutes each type of exercise. Responses require participants to indicate how many fifteen-minute periods they spend on each type of exercise in a typical week. From the responses a Leisure Score Index (LSI) can be calculated (9 × strenuous + 5 × moderate). Those with an LSI ≥ 24 are deemed active, whilst those below this are considered inactive. These data were collected to assess typical activity levels and ensure they were comparable between groups. Results from screening surveys were reviewed within 7 days of completion and all participants were emailed. Eligible participants were invited by email to attend the laboratory for the acute exercise intervention. Once a testing session was agreed, participants were sent a reminder 24–48 h prior to the session.

#### 2.5.2. Randomisation

Using a web-based random number generator, randomisation was stratified by group (Healthy Control, Medicated ADHD and Unmedicated ADHD) with a 1:1 ratio between allocation to cycling and Hatha yoga interventions. Randomisation was conducted with pre-set participant IDs prior to recruitment and screening. Additionally, the same randomisation method was used to allocate participants to one of two versions of the Iowa Gambling Task to prevent carry-over effects. This was not stratified by group.

#### 2.5.3. Intervention

Upon arrival at the laboratory, participants were given a pre-set participant ID, which was associated with the randomisation described above. They provided written consent and confirmed that they had not consumed caffeinated drinks or alcohol on the test day or smoked for at least three hours beforehand. Blood pressure was measured to ensure participants had healthy values and were able to exercise safely. The participants were required to undertake cognitive testing to measure core symptoms of ADHD at baseline and after the exercise intervention (see ‘Measures’). Immediately prior to exercising, resting heart rate was measured in all participants. For cycling, participants were required to complete a 2 min warm-up on a stationary exercise bike (Ultrasport F-Bike 300B Bike Trainer with Backrest, Castle Donnington, UK.) before cycling at a moderate intensity (70–80 RPM) for 10 min, measured using an indoor cycling mobile application (Motosumo, Herlev, Denmark) which provided the average RPM for the period. Those in the Hatha yoga condition completed a 2 min introduction and positioning activity before undertaking Hatha yoga for 10 min following an instructional video. This consisted of floor-based work using a yoga mat, but participants were provided with yoga blocks should they wish to use them at the points recommended in the video to make movements more comfortable. The yoga exercises were pitched at beginner levels and provided by a qualified yoga instructor. The exercises were designed to focus on shoulder mobility, and included seated forward fold and chest stretches, child’s pose and half down dog. Throughout the exercises, guided breathing instruction was given. The screen displaying the instructional video was lowered to floor level for comfort. The 10 min exercise duration was based on pilot work conducted in our laboratory and previous studies which indicated short duration (≤10 min) of aerobic exercise could be effective [50,51,53]. The Hatha yoga duration was matched to this. Immediately after exercise, heart rate was again measured to calculate exercise intensity, where the intended moderate intensity should increase heart rate to between 50–70% of the maximum [69], with the maximum calculated by subtracting age from 220 [70]. Additionally, participants were asked to rate their perceived exercise exertion using the Borg 6–20 Rating of Perceived Exertion (RPE) scale [71].

#### 2.5.4. Measures

The outcomes in this trial measured the core symptoms of ADHD: inattention, impulsivity and hyperactivity. These were collected using the tasks outlined below. Presentations of the tasks were randomised using a Latin square randomisation on the experimental platform Gorilla such that each participant completed the tasks in a random order before and after exercise.

Test of Variables of Attention (TOVA) Task: Individuals with ADHD show difficulties sustaining attention during effortful tasks and are inattentive to environmental cues, which can significantly impact daily functioning [72]. Several different tests can be used to measure attention, but those based on a Continuous Performance Task (CPT) paradigm have been found to be highly sensitive to the deficits found in ADHD, including adult populations [73,74,75,76]. The CPT found to best differentiate those with ADHD from healthy controls and individuals with other psychiatric conditions is the TOVA task, which aligns well with the current understanding of attention [77]. The TOVA task lasts for 22 min, which is longer than people with ADHD can typically stay vigilant for, making it a more sensitive test of this population than short CPTs [78]. Participants are required to respond to target (or ‘Go’) stimuli whilst inhibiting responses to non-target (‘No-Go’) stimuli. Stimuli flash up for a period of 100ms on the screen, with one stimulus presented every 2s. There are two phases of the task, from which different conclusions can be made: i) Phase 1—Go signals are infrequent, presented in just 22.5% of the trials; ii) Phase 2—Go signals are frequent, presented in 77.5% of the trials. The first phase provides information about inattention and the second about motor impulsivity [77,79]. Several different response parameters were measured. In the present study, omission errors and hit reaction times were collected from Phase 1 to provide measures of attention; *d* prime was calculated based on performance in this phase as a measure of response sensitivity related to attention. In Phase 2, commission errors were recorded as a measure of motor impulsivity, or the inability to inhibit a response. All participants received the same on-screen instructions before viewing practice screens, which showed example stimuli and indicated what response, if any, would be required for the stimulus shown. After viewing the examples, participants started the trials (650 trials). 

Delay Discounting Test (DDT): This task is a reliable and valid measure of temporal impulsivity that is elevated in those with ADHD relative to healthy controls [80]. The task involves presenting participants with several choices between hypothetical rewards now or at a point in the future. This process is repeated once for each delay with descending and ascending reward amounts and for several different future time points, e.g., 1 week, 2 weeks, 1 month, 3 months, 6 months and 1 year. The participants receive standardised on-screen instructions before starting the trials and the whole task (364 trials) takes less than 15 min to administer and can produce several parameters that can be used as a measure of temporal impulsivity (indifference points, discounting function, area under the curve, AUC). In the current study, we opted to compute the AUC because this variable is purely derived from the data and not based on assumptions about the form of the discount function [81,82]. A higher value for AUC indicates reduced impulsivity.

Iowa Gambling Task (IGT): One of the most frequently used and ecologically valid assessments of cognitive impulsivity is the Iowa Gambling Task (IGT), a computerised neuropsychological task in which participants are shown four virtual decks of cards (A, B, C and D) [83]. Differences in this task have been found in individuals with ADHD who exhibit riskier choices [84,85,86]. Participants receive standardised on-screen instructions and then complete 100 trials. For each trial, they choose a card; they can win or lose virtual money. Two decks give large gains and large losses and are considered riskier choices, whereas the other two decks give small gains and small losses. High cognitive impulsivity is shown by more frequent choices of the riskier decks. This can be measured over all 100 trials, but the early trials in the task (~40 trials) are typically associated with a period of learning where the individuals have no explicit knowledge to support their decisions [87]; therefore, more accurate data may be collected from the latter phase. In this case, we used data from the final 40 trials. The whole task takes approximately 15 min to administer. Given that participants could recall which decks were associated with specific gains and losses between the baseline and post-exercise time points, we counterbalanced the use of two different versions of the task, where the risky vs non-risky decks were not the same. Two measures were made from the final 40 trials; firstly, the percentage of risky choices and secondly the net score, calculated by subtracting risky choices from choices, such that a more negative net score indicates more risky decisions.

Actigraphy Measurements: During completion of the cognitive tests, participants were required to wear an actigraph (Micro Motionlogger. Ambulatory Monitoring Inc., New York, USA) on their non-dominant wrist to measure movement. These devices can differentiate participants with ADHD from those without [88,89,90]. The actigraph provides indices of movement frequency (zero crossing mode, ZCM) and intensity (proportional integrating measure, PIM).

#### 2.5.5. Changes to Protocol

The inclusion criteria noted in the protocol for those with ADHD differed slightly from the final inclusion criteria. In the protocol, all individuals with ADHD had to be free from all other psychiatric conditions. However, following the NHS Ethics application, we were advised to include depression and anxiety provided individuals were not receiving medication for these. Additionally, in the protocol we aimed to have unmedicated participants drug-free for six months. We amended this to three months, in line with other work, to increase the number of eligible participants. The planned sample size for this study was based on a 3 × 2 × 2 factorial design with Group (HC, medicated ADHD, unmedicated ADHD), Time (pre, post) and Exercise (cycling, Hatha yoga). Although randomisation was stratified by these three groups, due to difficulties recruiting participants with ADHD, particularly those who were unmedicated, during the COVID-19 pandemic we were unable to recruit sufficient individuals for this design to be adequately powered (≥0.80). To address this, we opted to analyse the data using a 2 × 2 × 2 design (Group—Healthy Control, ADHD; Time—Pre, Post; Exercise—Cycling, Hatha Yoga). Prior to making this decision, we compared ASRS scores between medicated and unmedicated individuals to check if there were any significant symptomology differences between the groups that would advise against combining them. We found no such differences (ASRS total t(80) = 0.551, *p* = 0.584, ASRS-Inattention t(80) = −0.024, *p* = 0.981; ASRS-Hyperactivity-Impulsivity t(80) = 0.747, *p* = 0.547). In addition, previous work suggests medication does not impact the effects of exercise [55]. However, as indicated below, several covariates required use of an ANCOVA. To achieve minimally acceptable power (≥0.80) for a small effect size (0.2), an ANCOVA with three covariates requires a sample size of 128, which we did exceed (except for measures of hyperactivity, where *N* = 127).

#### 2.5.6. Statistical Analysis

All analyses were performed with a two-tailed significance value of 5% and data were checked for normality using histograms, measures of skewness and kurtosis and the Kolmogorov-Smirnov normality test. Descriptive data (M ± SD or N/%) were used to characterise the sample in terms of demographic, clinical and heart rate variables. Group comparisons were made with *t*-tests and chi-square analyses. To assess exercise fidelity (i.e., that aerobic cycling really was raising heart rate), *t*-tests were used on heart rate pre- and post-exercise and descriptive data were reported for the Borg RPE measure. The relationship between outcome measures and clinical characteristics was assessed using Pearson’s *r* with pre-exercise data to provide additional context. Finally, the main analyses were conducted on an intention-to-treat basis, where any post-exercise missing data were replaced with pre-exercise data for an individual, i.e., the ‘last observation carried forward’ approach. This approach was deemed appropriate given that all testing takes place within a single two-hour period and therefore, extraneous variables or illnesses are unlikely to impact and the resultant analyses can be deemed plausible [91]; the amount of missing data was minimal, meaning this approach will not undermine the effects of the intervention. Details of per-protocol and intention-to-treat sample sizes are provided in Supplementary Table S1. Note that actigraphy measures were collected from fewer participants due to delays in receiving a replacement adaptor for the actigraph, which was delayed during import to the UK, meaning the equipment could not be used for a brief period. Examination of the data revealed that gender, age and pre-exercise heart rate differed between the groups and as such these were included in the analysis as covariates. A 2 × 2 × 2 ANCOVA was conducted for all outcome measures. Any significant interactions were explored with independent and paired-sample *t*-tests.

## 3. Results

### 3.1. Study Population

**Figure 1:**
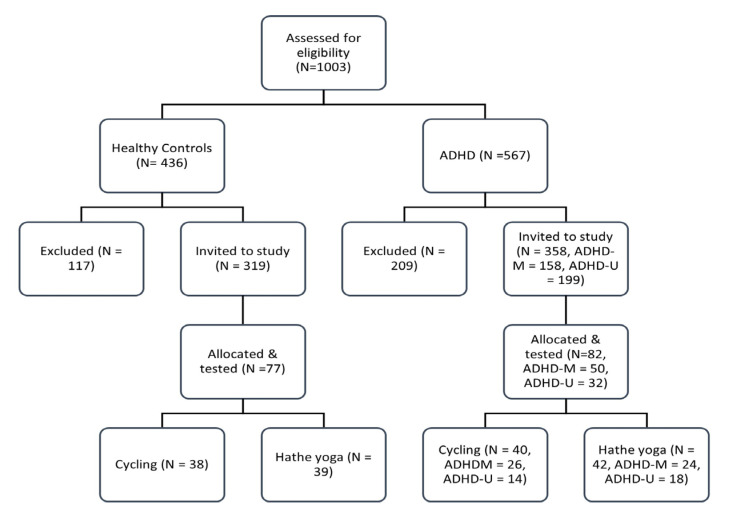
Flow diagram showing screening and allocation of the different groups. ADHD-M indicates medicated with psychostimulants whilst ADHD-U indicates medication free. Although the two groups were combined in the final analysis to increase statistical power, the initial randomisation was stratified according to three groups (HC, ADHD-M and ADHD-U).

In total, 1003 potential participants were screened, of which 326 were excluded for not meeting the eligibility criteria. Within the HC group, this was largely because of high ASRS scores (*N* = 108, 92% of HC exclusions) and five fell outside the required age range (*N* = 5, 4%) or reported other conditions requiring exclusion (*N* = 2, 2%). Within the ADHD groups, few exclusions were made for low ASRS scores (*N* = 17, 8% of ADHD exclusions) with most exclusions being due to low medication adherence (*N* = 87, 42%) or the presence of comorbid conditions (*N* = 53, 25%). Other reasons for exclusion included use of medications other than stimulants (*N* = 18, 9%) and being outside the required age range (*N* = 33, 16%). All those eligible were invited to attend. A total of 159 adults (77 HC, 82 ADHD) were randomly allocated to the aerobic cycling or Hatha yoga condition (Figure 1). 

Demographic and clinical characteristics of the two groups are detailed in Table 1. As indicated, there were significantly more females with ADHD than the HC group. Additionally, the ADHD group had a significantly higher mean age, albeit differing by less than 4 years. There was also a significantly higher resting heart rate in those with ADHD. Given that use of stimulants is associated with a higher resting heart rate [92], this effect is likely to be driven by use of stimulant medication in over half of this group (*N* = 50 medicated, *N* = 32 unmedicated) with adherence to medication at 89% (*SD* = 119%). This was confirmed by a one-way ANOVA, which indicated significant differences between the medicated participants with ADHD and both unmedicated and HC (*p* <.001), but no other group differences. As expected, the HC group had significantly lower ASRS scores.

### 3.2. Exercise Fidelity

To ensure that the cycling and Hatha yoga conditions were altering heart rate (HR) as expected, we used paired sample *t*-tests to examine HR before and after both types of exercise. As expected, cycling resulted in a significant increase from pre- (*M* ± *SD,* 72.03 ± 10.65) to post-exercise HR (109.55 ± 21.23; *t*(77) = 16.11, *p* < 0.001). In contrast, yoga did not significantly change HR from pre- (70.59 ± 10.83) to post-exercise (69.94 ± 11.50; *t*(77) = 0.838, *p* = 0.405). The average RPM for the duration of cycling was 77.00 (7.04), as required in the instructions to participants. Furthermore, Borg Rating of Perceived Exertion (RPE) showed that the most frequent perception of cycling was ‘Somewhat hard’ or ‘Hard heavy’ (both 28.2%). In contrast, yoga was most associated with ‘No exertion’ (44.40%), therefore demonstrating participants perceived the cycling as more strenuous as well as experiencing increased heart rate. To provide a more objective measure of intensity, we used the moderate activity definition where this intensity of exercise should increase heart rate to between 50–70% of the maximum [69], with the maximum calculated by subtracting age from 220 [70]. The intensity for cycling was 53% and 59% of the maximum HR in the HC and ADHD groups, respectively, and was less intense for yoga (35% and 38% for HC and ADHD, respectively, as shown in Supplementary Table S2). Given the baseline differences in HR between the groups, we examined heart rate changes within and between groups and found no differences in the exercise-induced heart rate increases between HC and ADHD groups (Supplementary Table S2).

### 3.3. Treatment Outcomes

As indicated in the statistical analysis section, analysis was conducted on an intention-to-treat basis with missing post-data replaced with pre-data. Note that all participants completed all tasks, and missing data were due to errors in task saving. Data loss was minimal, with ≤2 participants’ data missing within either the ADHD or HC groups for specific measures (see Supplementary Table S1). Descriptive data by group and exercise type for all measures are shown in Table 2. Prior to the main analyses, bivariate correlations between the outcome measures and the Adult Self-Report Scale (ASRS) scores were calculated (Supplementary Tables S3 and S4). As would be expected, the total score, and inattention (IA) and hyperactive-impulsive (HI) sub-scores were significantly correlated with each other in both HC and ADHD groups (*p* < 0.01). Additionally, the different attention outcome measures (omission errors, hit reaction time and d prime) significantly correlated with each other in HC (*p* < 0.01) but within the ADHD group, only omis-sion errors and d prime, and d prime and hit reaction time correlated (*p* < 0.01). The two measures of cog-nitive impulsivity (net score and percentage risky decisions) were also significantly correlated in both groups, as were the two measures of hyperactivity (frequency and movement intensity, *p* < 0.01). Within the HC group, there were no significant correlations between cognitive or behavioural measures of differ-ent constructs, but the ASRS total and ASRS-IA correlated with the measure of temporal impulsivity (AUC *p* < 0.01). Within the ADHD group, performance on tasks did correlate; omission errors correlated significantly with measures of cognitive and temporal impulsivity (*p* < 0.01), hit reaction time correlated with temporal impulsivity (*p* < 0.01), d prime correlated with commission errors (*p* < 0.01) and, finally, commission errors correlated with measures of cognitive impulsivity (*p* < 0.01). These correlations sug-gest some significant relationship between cognitive measures in those with ADHD that are not present in those without. The ASRS total and ARSR-IA significantly correlated with both movement intensity and frequency.

#### 3.3.1. Inattention

After controlling for the covariates of gender, age and resting heart rate, for Hit Reaction Time, there was no significant main effect of Time (*F*(1, 150) = 0.05, *p* = 0.822, *ηp*^2^ = 0.000), Exercise (*F*(1, 150) = 0.02, *p* = 0.889, *ηp*^2^ = 0.000) or Group (*F*(1, 150) = 2.13, *p* = 0.146, *ηp*^2^ = 0.014). There was, however, a significant Time x Group interaction (*F*(1, 150) = 4.11, *p* = 0.044, *ηp*^2^ = 0.027). Examination of the data with independent *t*-tests indicates that prior to exercise, those with ADHD had significantly longer reaction times than those without ADHD (*t*(135.52) = 2.33, *p* = 0.021). However, after exercise the groups were comparable (*t*(157) = 0.07, *p* = 0.472). Paired sample *t*-tests indicated that whilst the ADHD group did not significantly change their reaction time after exercise (*t*(81) = 0.85, *p* = 0.396), there was a significant increase in reaction time in the HC group (*t*(76) = 3.15, *p* = 0.002). For omission errors, there were no significant main effects (Time *F*(1, 150) = 0.20, *p* = 0.653, *ηp*^2^ = 0.001; Exercise *F*(1, 150) = 0.18, *p* = 0.671, *ηp*^2^ = 0.001; Group *F*(1, 150) = 2.27, *p* = 0.134, *ηp*^2^ = 0.015) or two- or three-way interactions (*p* ≥ 0.249). Finally, after controlling for covariates, there were no significant main effects (Time *F*(1, 150) = 0.93, *p* = 0.326, *ηp*^2^ = 0.006; Exercise *F*(1, 150) = 0.12, *p* = 0.735, *ηp*^2^ = 0.001; Group *F*(1, 150) = 0.57, *p* = 0.451, ηp2 = 0.004) or two- or three-way interactions (*p* ≥ 0.075) for *d* prime.

#### 3.3.2. Temporal Impulsivity

**Figure 2:**
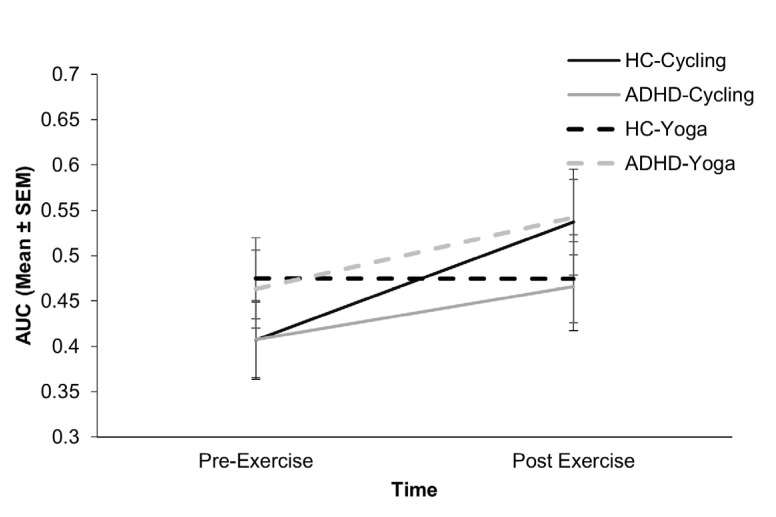
Temporal impulsivity, measured by the Area Under the Curve (AUC) in the Delay Discounting Task demonstrated that cycling exercise increased the AUC for healthy controls (HC-Cycling) and those with ADHD (ADHD-Cycling), indicative of reduced impulsivity. However, yoga only improved temporal impulsivity in the ADHD group (ADHD-Yoga) and not the control group (HC-Yoga).

After controlling for gender, age and pre-exercise heart rate, there was no significant main effect of Time (*F*(1, 142) = 3.08, *p* = 0.081, *ηp*^2^ = 0.021), Exercise (*F*(1, 142) = 0.50, *p* = 0.498, *ηp*^2^ = 0.003) or Group (*F*(1, 142) = 0.01, *p* = 0.815, *ηp*^2^ = 0.000) on the AUC. There were also no significant two-way interactions (*p* ≥ 0.153). However, there was a significant Time x Exercise x Group interaction (*F*(1, 142) = 4.79, *p* = 0.03, *ηp*^2^ = 0.033) (Figure 2). Paired sample *t*-tests indicate that cycling increased the AUC for both HC (*t*(35) = 2.17, *p* = 0.037) and those with ADHD (*t*(33) = 2.66, *p* = 0.012). Similarly, yoga increased AUC in those with ADHD (*t*(41) = 3.03, *p* = 0.004) but not for HC (*t*(38) = 0.23, *p* = 0.824). Given that higher AUC is indicative of reduced impulsivity, this indicates that cycling and yoga can be beneficial in those with ADHD, whilst only cycling is effective for HC.

#### 3.3.3. Motor Impulsivity

After controlling for gender, age and pre-exercise heart rate, there was no significant main effect of Time (*F*(1, 149) = 0.79, *p* = 0.374, *ηp*^2^ = 0.005), Exercise (*F*(1, 149) = 0.27, *p* = 0.602, *ηp*^2^ = 0.002) or Group (*F*(1, 149) = 2.16, *p* = 0.144, *ηp*^2^ = 0.014) on commission errors. Also, there were no significant two or three-way interactions (*p* ≥ 0.168).

#### 3.3.4. Cognitive Impulsivity

For the percentage of risky decisions in the last 40 trials, after controlling for gender, age and pre-exercise heart rate, there was no significant main effect of Time (*F*(1, 150) = 2.70, *p* = 0.102, *ηp*^2^ = 0.018), Exercise (*F*(1, 150) = 1.00, *p* = 0.319, *ηp*^2^ = 0.007) or Group (*F*(1, 150) = 0.01, *p* = 0.912, *ηp*^2^ = 0.000). There were no significant two or three-way interactions (*p* ≥ 0.103). The Net Score for the last 40 trials revealed similar null effects (Time *F*(1, 150) = 2,71, *p* = 0.100, *ηp*^2^ = 0.018), Exercise (*F*(1, 150) = 0.73, *p* = 0.394, *ηp*^2^ = 0.005), Group (*F*(1, 150) = 0.06, *p* = 0.809, *ηp*^2^ = 0.000), interactions *p* ≥ 0.103).

#### 3.3.5. Motor Activity

For frequency of movement, there was no significant main effect of Time (*F*(1, 118) = 0.66, *p* = 0.419, *ηp*^2^ = 0.006) or Exercise (*F*(1, 118) = 0.40, *p* = 0.530, *ηp*^2^ = 0.003) but there was a significant main effect of Group (*F*(1, 118) = 4.53, *p* = 0.035, *ηp*^2^ = 0.037), with those with ADHD more active than those without. Additionally, there was a significant Time x Group interaction (*F*(1, 118) = 8.49, *p* = 0.004, *ηp*^2^ = 0.067). There were no other significant two- or three-way interactions (*p* ≥ 0.095). Examination of the data with independent *t*-tests indicates that both prior to (*t*(125) = −0.169, *p* = 0.093) and after (*t*(125) = −0.193, *p* = 0.847) exercise, HC and ADHD activity levels were similar. However, paired sample *t*-tests indicate that whilst the ADHD group showed no significant change after exercise (*t*(58) = 0.26, *p* = 0.800), the HC showed a significant increase in movement after exercise (*t*(67) = 3.18, *p* = 0.002). For the intensity of movement, there were no significant main effects (Time (*F*(1, 118) = 0.14, *p* = 0.715, *ηp*^2^ = 0.001), Exercise (*F*(1, 118) = 0.60, *p* = 0.440, *ηp*^2^ = 0.005) or Group (*F*(1, 118) = 3.25, *p* = 0.074, *ηp*^2^ = 0.027). However, there was a significant Time x Group interaction (*F*(1, 118) = 4.81, *p* = 0.030, *ηp*^2^ = 0.039). Examination of the data revealed that prior to exercise, the ADHD group had significantly greater intensity of movement (*t*(125) = 2.34, *p* = 0.021). However, after exercise this difference was no longer significant (*t*(125) = 0.64, *p* = 0.522). Paired sample *t*-tests revealed that the ADHD group again did not change after exercise (*t*(58) = 1.48, *p* = 0.145) whilst for the HC group movement intensity increased (*t*(67) = 5.24, *p* <.001).

## 4. Discussion

The aim of this study was to establish the effects of aerobic cycling and mind-body Hatha yoga exercise on ADHD symptomology in adults with the condition, in contrast to a HC group. The data demonstrated a positive effect of exercise on temporal impulsivity, in which cycling reduced impulsivity for both groups, whilst yoga additionally reduced impulsivity within the ADHD group. There were no effects of exercise on any measure of attention, cognitive or motor impulsivity, or movement in those with ADHD. However, exercise did increase hit reaction time, a measure of attention, in the HC group, meaning that after exercise the two groups were comparable despite the ADHD group having longer reaction times pre-exercise. Similar effects were seen for movement frequency and intensity with increases in these measures after exercise in the control group, leaving them statistically similar to those with ADHD. It is also noteworthy that scores on the ASRS, which is a validated measure of ADHD traits, did not always correlate with the objective measures of attention, impulsivity and hyperactivity. For the HC group, there was only a correlation with temporal impulsivity, whilst for those with ADHD, ASRS measures correlated with some measures of attention, cognitive and temporal impulsivity and movement, but not motor impulsivity.

No previous research has looked at delay discounting in those with ADHD before and after exercise; however, there is some indication that physical activity, albeit when maintained for some time, can delay discounting in healthy individuals [93]. In addition, research on individuals experiencing pain [94] or in elderly populations [95] suggests that exercise is associated with reduced temporal impulsivity, albeit not measured with the delay discounting task used in the present study. Interestingly, in the current study, temporal impulsivity was improved by cycling for both groups, but yoga was only effective for those with ADHD. The basis for this change is uncertain. However, evidence suggests that the catecholamine systems may be heightened post-exercise [25,27,28], and several studies have now reported increased dopamine after meditation or yoga in healthy adults [27,28]. Given the relationship between dopamine and reward, increased dopamine could adjust the value ascribed to delayed rewards [96], reducing temporal impulsivity. The extent of the change in dopamine may underlie the differential effects of exercise type on HCs when compared to those with ADHD.

Although no previous work has examined cognitive impulsivity before and after exercise in ADHD, the lack of effects of exercise on attention and motor impulsivity in the ADHD group is at odds with previous work, most notably in two studies on adults examining attention and response inhibition after cycling [58,59]. However, in both cases the duration of exercise was longer than that used in the current study, although intensity was reportedly similar. Additionally, for response inhibition, positive effects were only found for those who performed significantly worse initially [59]; in the present study, those with ADHD were not worse than the HC group at baseline. Despite the work by Mehren and colleagues indicating positive effects in adults with ADHD, other researchers have failed to find improvements in cognition after cycling [33], suggesting that the effects are not reliably found. The work by Fritz and O’Connor [33] was also the only study to have measured hyperactivity using an accelerometer and found no significant impact of exercise on movement in those with ADHD, aligning with the results of the current study. 

Perhaps more surprising than the lack of effects in those with ADHD are the effects on HCs. Based on the previously reported benefits of exercise on cognition in healthy participants [23,24], it may be surprising that the HC group showed an increase in reaction time after exercise on the Test of Variables of Attention (TOVA) task. However, this is not inconsistent with previous work. Silva et al. [50] conducted a study in children with and without ADHD and found after acute exercise (5 min of running), the control group experienced a 42% decline in attention measures. In addition, recent work in university students with and without ADHD compared performance on a Continuous Performance Task (CPT) before and during exercise and found that, whilst the ADHD group improved, those without ADHD performed worse during exercise [97]. The authors suggest that when at rest, the attentional system in control participants is likely to be functioning optimally and that splitting attention between exercise and the CPT results in impaired performance. Whilst this may explain their results, it seems unlikely here given that we saw the decline post-exercise rather than during it. However, if the catecholamine systems are heightened post-exercise [25,27,28], in HCs this could result in too much dopamine, disrupting attentional processes according to the inverted U theory of dopamine and cognition [98]. In addition to mixed effects on the groups for cognition, actigraph data indicated that the HC group increased in movement after exercise. If the exercise had increased dopamine levels as we tentatively speculate, this could explain the effects of movement in this group. Given that we did not measure dopamine in this study, this would need to be directly investigated in future work. 

This study is the first to examine the effects of acute exercise on measures of all core symptoms of ADHD in an adult population. However, it is not without limitations. Firstly, participants could not be blinded to their condition, given the nature of the intervention. Additionally, researchers were not blinded because they monitored exercise, although they were not responsible for measuring outcomes, with all data being collected through computerised tests. Secondly, arguably the fact that the ADHD sample was female-dominated is a limitation and means that results may not generalise to a more gender-balanced cohort. ADHD is more prevalent in boys than girls, although the gender difference in prevalence reduces considerably in adulthood [99] but not to the extent that females outnumber males. Furthermore, some have argued that there are differences in cognitive function between males and females with ADHD in adulthood [100], and others have said that the genders are more similar than different [101]. Therefore, a more balanced sample would be ideal. Despite this, it is noteworthy that females can be under-represented in ADHD research [102], meaning that this limitation may also be seen as a strength. Also relating to the demographic characteristics of the sample, on average both groups had over 5 years post-compulsory education. Whilst around 50% of young people in the UK attend university, this could indicate our sample was more highly educated than is typical. Thirdly, we did not include a no-exercise control condition, which could be viewed as a limitation. The cognitive tests that participants completed before and after testing are lengthy and it is possible that some effects observed were not related to exercise but merely fatigue. For example, the increase in reaction times or movement observed in the HC group could be attributed to fatigue. However, this seems unlikely because these effects were not seen in the ADHD group, who would be as susceptible or more so to cognitive fatigue. Including a no-exercise condition in future studies would allow this possibility to be examined. Fourthly, we did not measure heart rate during exercise, meaning the HR measures may not reflect the entire 10 min period. This could have been achieved, for example, with a heart rate chest strap, to allow constant monitoring rather than simply pre- and post-exercise. This would also have allowed the rate of change of the heart rate to be considered, which is likely to vary with fitness level. As such, future studies should consider if this can be achieved without discomfort in both exercise conditions. Our cycling condition, whilst significantly increasing heart rate and being reported as ‘Somewhat hard’ or ‘Hard heavy’, was only just within the range of the heart rate increase associated with moderate exercise [69], which could mean that the lack of effects are due to the intensity of the exercise. This seems unlikely given some effects were found, and because previous studies have indicated intensity does not necessarily impact effects [54] and that as little as five minutes of exercise may be beneficial [50,51]. It should also be noted that whilst overall participants showed an elevated heart rate within the moderate level, this was not individualised. A more precise approach would have been to ensure HR increases were personalised. Additionally, due to the challenges of recruiting participants during COVID-19, we had to combine our medicated and unmedicated groups. Although the medicated and unmedicated groups did not differ in terms of ASRS scores suggesting similar severity, this combination prevented us from distinguishing between standalone and adjunct effects of medication. When combined, we required a total sample size of 128 to meet the minimally required statistical power. This was exceeded on all measures of attention and impulsivity, but we were slightly below this for measures of hyperactivity (N = 127). Finally, we did not confirm ADHD diagnosis as part of the study; rather, we asked participants to confirm they had received a diagnosis from a clinician and they completed the ASRS to ensure that they were only included if they scored over a pre-determined threshold [65]. Similarly, we did not confirm a lack of diagnosed conditions in the HC participants but asked them to self-report that they had not received any diagnosis of ADHD or other excluded condition and to complete the ASRS. It is therefore possible some individuals had undiagnosed conditions or gave false responses.

Despite these limitations, there are several strengths of the study. Firstly, we employed a randomised controlled design, which is widely considered the gold standard. Secondly, our groups were matched for activity levels which minimised the impact of group level differences in fitness. Thirdly, we included commonly found comorbidities to ensure an ecologically valid sample, given the higher comorbidity in adult ADHD [67]. Fourthly, the exercise interventions tested were suitable for adults with differing levels of fitness and are short, making them feasible to include in routines. Fifthly, we used objective computerised tests of ADHD symptoms rather than relying extensively on self-report measures, as is common. Finally, although the sample size did not reach our initial planned size, it is still much larger than most previous studies in this area, as discussed in recent systematic reviews [22,35,37]. As such, this research makes a significant contribution to the current research literature which has seen adults and mind-body non-aerobic exercise neglected. 

## 5. Conclusions

In conclusion, to our knowledge, this is the first study to adopt a fully randomised controlled trial design to examine the effects of two types of exercise in adults with ADHD. The results indicate that as little as ten minutes of cycling or Hatha yoga may be beneficial for individuals with ADHD, reducing their temporal impulsivity. Furthermore, the results suggest that effects of healthy controls also warrant further investigation, and that exercise may not always be beneficial to cognition in these individuals.

## Figures and Tables

**Table 1:** Baseline demographic and clinical characteristics of the two groups.

	HC (*N* = 77)	ADHD (*N* = 82)	*t* or *Χ*^2^	Significance
Demographic variable; *N* (%)				
Female gender	41 (53)	68 (83)	16.23	<0.001
Right-handed *	70 (91)	73 (88)	0.436	0.509
Active LSI	67 (87)	70 (85)	0.090	0.764
Age (years) M (SD)	23.01 (4.29)	26.62 (5.26)	3.576	<0.001
Education (years) M (SD)	5.36 (3.24)	5.32 (3.06)	0.093	0.926
Resting Heart Rate (bpm) M (SD)	67.97 (8.56)	74.44 (11.57)	4.03	<0.001
Clinical Characteristics M (SD)				
ASRS Total	21.29 (9.17)	54.70 (7.68)	24.96	<0.001
ASRS-Inattention	11.97 (5.10)	29.73 (3.86)	24.86	<0.001
ASRS-Hyperactivity-Impulsivity	9.29 (5.00)	24.99 (5.56)	18.67	<0.001

* Chi-square compared only right- and left-handed individuals because only one participant reported being ambidextrous.

**Table 2:** Descriptive statistics for Healthy Controls and those with ADHD, before and after cycling and yoga. All scores expressed as M ± SD.

	Aerobic Cycling Exercise				Mind Body Hatha Yoga Exercise			
	Healthy Controls		ADHD		Healthy Controls		ADHD	
	Pre	Post	Pre	Post	Pre	Post	Pre	Post
**Inattention**								
Omission Errors	2.38 (4.26)	5.63 (13.38)	3.91 (7.18)	5.41 (15.37)	2.33 (5.43)	6.01 (12.75)	6.45 (15.19)	5.06 (10.49)
Hit Reaction Time	418.56 (59.94)	437.62 (93.48)	440.82 (96.07)	429.63 (83.35)	404.71 (60.64)	433.45 (109.80)	442.15 (101.97)	439.15 (73.98)
d Prime	0.10 (1.24)	−0.49 (1.38)	−0.11 (1.20)	0.02 (1.20)	−0.30 (1.35)	−0.02 (1.40)	−0.19 (1.43)	−0.04 (1.32)
**Motor impulsivity**								
Commission Errors	3.36 (3.07)	3.42 (2.80)	4.80 (4.74)	3.76 (3.77)	4.73 (10.58)	2.97 (2.73)	4.49 (5.66)	4.28 (5.50)
**Cognitive Impulsivity**								
Net Score (40)	−3.34 (17.41)	−10.21 (20.73)	−6.65 (21.03)	−1.45 (22.09)	−7.95 (20.39)	−8.90 (22.37)	−5.42 (21.95)	−8.38 (23.56)
% Risky Decisions	53.29 (22.26)	61.58 (27.27)	58.31 (26.28)	51.81 (27.61)	58.40 (26.05)	61.12 (27.97)	56.79 (27.44)	59.98 (29.20)
**Temporal Impulsivity**								
AUC	0.41 (0.25)	0.54 (0.35)	0.41 (0.25)	0.47 (0.25)	.47 (0.27)	0.47 (0.27)	0.46 (0.27)	0.54 (0.26)
**Hyperactivity**								
Motor frequency	2.89 (1.64)	3.17 (1.46)	3.86 (1.52)	3.58 (1.75)	2.76 (1.72)	3.43 (2.21)	2.86 (1.60)	3.19 (1.69)
Motor intensity	27.55 (17.73)	38.42 (21.69)	41.25 (21.25)	47.30 (38.99)	29.13 (21.40)	39.09 (26.66)	33.21 (21.37)	4.13 (23.37)

## Data Availability

Data are available on reasonable request from the corresponding author.

## Supplementary Materials

The following supporting information can be downloaded at: https://www.mdpi.com/article/10.3390/bs13020129/s1, Table S1: Sample sizes for analysis indicating the sample size for per-protocol (PP) and intention-to-treat (ITT) where missing data were replaced by pre-exercise (baseline values), Table S2: Heart rate and Borg Rating of Perceived Exertion (RPE) for the different forms of exercise by participants group, Table S3: Correlation between ASRS measures and cognitive and behavioural measures prior to exercise in the HC group. * *p* < 0.05, ** *p* < 0.01, Table S4: Correlation between ASRS measures and cognitive and behavioural measures prior to exercise in the ADHD group. * *p* < 0.05, ** *p* < 0.01.
