# Supplementary material for: The Effects of Different Exercise Approaches on Attention Deficit Hyperactivity Disorder in Adults: A Randomised Controlled Trial
Source: Behav Sci (Basel). 2023 Feb 2;13(2):129. doi: 10.3390/bs13020129 (PMC9952527; doi:10.3390/bs13020129)
Supplement: Supplementary file 1 [file behavsci-13-00129-s001.zip › behavsci-2183601-supplementary.pdf]

Table S1. Sample sizes for analysis indicating the sample size for per-protocol (PP) and intention-to-treat (ITT) where missing data was replaced by pre-exercise (baseline values).

**Measure**	**HC**				**ADHD**					
	**Cycling**		**Yoga**		**Cycling**		**Yoga**		**Total**	
	**PP**	**ITT**	**PP**	**ITT**	**PP**	**ITT**	**PP**	**ITT**	**PP**	**ITT**
Inattention										
Omission Errors	38	38	38	39	39	40	42	42	157	159
Hit Reaction Time	38	38	38	39	39	40	42	42	157	159
d′ Prime	37	38	38	39	40	40	42	42	157	159
Motor impulsivity										
Commission Errors	37	37	38	39	39	40	42	42	156	158
Cognitive Impulsivity										
Net Score (40)	37	38	37	39	38	40	40	42	152	159
% Risky Decisions	37	38	37	39	38	40	40	42	152	159
Temporal Impulsivity										
AUC	35	36	37	39	33	34	40	42	145	151
Hyperactivity										
Motor frequency	33	33	35	35	26	27	32	32	126	127
Motor Intensity	32	33	35	35	26	27	31	32	124	127

Table S2. Heart rate and Borg Rating of Perceived Exertion (RPE) for the different forms of exercise by participants group.

Measure	Aerobic Cycling Exercise		Mind Body Hathe Yoga Exercise	
	HC (*N* = 38)	ADHD (*N* = 40)	HC (*N* = 39)	ADHD (*N* = 40)
Heart Rate M (SD)				
Pre-exercise	69.10 (8.12)	74.83 (12.05)	66.63 (8.92)	74.35 (11.23)
Post-exercise	103.45 (20.93)	115.35 (20.08)	66.55 (10.55)	73.15 (11.55)
Within group comparison	<.001	<.001	.939	.313
ΔHR	34.34 (19.39)	40.52 (21.43)	-0.08 (6.33)	-1.20 (7.42)
Between group comparison		.186		.476
Percentage of Max HR M (SD)	52.69 (10.25)	59.40 (10.03)	33.94 (5.25)	38.09 (5.89)
Between group comparison		.002		<.001
RPE N (%)				
No exertion		1 (2.5)	14 (43.6)	19 (45.2)
Extremely Light	1 (2.6)	2 (5.0)	11 (28.2)	5 (11.9)
Very Light	3 (7.9)	4 (10)	2 (5.1)	6 (14.3)
Light	8 (21.1)	4 (10)	5 (12.8)	6 (14.3)
Somewhat hard	11 (28.9)	11 (27.5)	3 (7.7)	1 (2.4)
Hard Heavy	12 (31.6)	10 (25.0)		
Very Hard	3 (7.9)	6 (15)		
RPE M (SD)	13.1 (2.36)	13.1 (2.97)	7.80 (2.27)	7.69 (2.10)

Table S3. Correlation between ASRS measures and cognitive and behavioural measures prior to exercise in the HC group. * p<.05, ** p<.01

Measure	ASRS Total	ASRS IA	ASRS HI	Omission Errors	Hit Reaction Time	d′ Prime	Commission Errors	Net Score (40)	% Risky Decisions	AUC	Motor frequency
ASRS IA	.909**										
ASRS HI	.905**	.647**									
Omission Errors	-.102	-.083	-.099								
Hit Reaction Time	.072	.050	.085	.323**							
d′ Prime	-.077	-.092	-.040	.324**	.146						
Commission Errors	.123	.032	.193	.122	.224	-.077					
Net Score (40)	-.084	-.121	-.032	.069	.047	.198	.051				
% Risky Decisions	.102	.130	.054	-.064	-.020	-.173	-.036	-.947**			
AUC	-.282*	-.358**	-.164	-.057	-.122	-.119	-.109	.044	-.027		
Motor frequency	.057	.062	.043	.076	-.062	-.079	-.090	.130	-.089	.051	
Motor intensity	-.027	-.019	-.036	.130	-.063	-.011	-.139	.230	-.209	.154	.793**

Table S4. Correlation between ASRS measures and cognitive and behavioural measures prior to exercise in the ADHD group. * p<.05, ** p<.01

Measure	ASRS Total	ASRS IA	ASRS HI	Omission Errors	Hit Reaction Time	d′ Prime	Commission Errors	Net Score (40)	% Risky Decisions	AUC	Motor frequency
ASRS IA	.731**										
ASRS HI	.878**	.317**									
Omission Errors	0.018	0.003	0.021								
Hit Reaction Time	0.121	0.156	0.057	0.055							
d′ Prime	-0.078	0.071	-0.161	.543**	.285**						
Commission Errors	0.154	0.007	0.204	-0.077	-0.087	-.242*					
Net Score (40)	-0.114	0.044	-0.181	-.246*	0.026	0.035	-.248*				
% Risky Decisions	0.114	-0.044	0.181	.246*	-0.026	-0.035	.248*	-1.000**			
AUC	-0.024	0.030	-0.052	.290*	-.376**	-0.044	0.049	-0.174	0.174		
Motor frequency	.363**	.388**	0.215	-0.005	0.205	-0.126	0.076	0.014	-0.014	0.064	
Motor intensity	.381**	.380**	0.248	-0.094	0.053	-0.241	0.113	0.049	-0.049	0.076	.773**
